# The Efficacy of Ayurvedic Herbs in the Prevention and Treatment of Inflammatory Bowel Disease: A Scoping Review

**DOI:** 10.7759/cureus.84410

**Published:** 2025-05-19

**Authors:** Sakshika Vakiti, Lessie Farriss, Hasrat Mehta, Meghana Madaram, Diana Ferrigno, Rita Shoukry, Jessica Arango Hipsley, Bona Lee, Lian Atlas-Grayton, Celia Carlisano, Stephanie N Petrosky

**Affiliations:** 1 Osteopathic Medical School, Nova Southeastern University Dr. Kiran C. Patel College of Osteopathic Medicine, Clearwater, USA; 2 Nutrition, Nova Southeastern University Dr. Kiran C. Patel College of Osteopathic Medicine, Clearwater, USA

**Keywords:** ayurveda, colitis, crohn’s, herbs, inflammatory bowel disease

## Abstract

Inflammatory bowel disease (IBD) is a chronic inflammatory condition of the gastrointestinal tract with no known cure in Western medicine. While steroids and other anti-inflammatory treatments help manage symptoms, Ayurvedic herbs and spices offer a holistic approach rooted in Ayurveda, an ancient Indian medical system that aims to balance life’s forces (doshas) within the mind, body, and soul. Although certain Ayurvedic herbs have demonstrated anti-inflammatory properties, a comprehensive review of their efficacy in IBD treatment remains limited. This study evaluates the effectiveness of Ayurvedic herbal extracts and formulations in the treatment and prevention of IBD through a review of literature from January 2003 to September 2023 across Embase, PubMed, and Cochrane Central. A total of 25 full-text articles were analyzed, with data compiled based on study characteristics and relevance to the research question. Findings indicate macroscopic, microscopic, and clinically significant benefits, including reduced mucus in stool, improved colonic weight retention, and decreased inflammatory markers such as tumor necrosis factor-α (TNF-α), interleukin-2 (IL-2), prostaglandin E2 (PGE2), and endothelium-dependent relaxation (EDR). Clinically, patients reported fewer urgent bowel movements and an improved sense of well-being. These results suggest that Ayurvedic herbs may serve as a complementary or alternative therapy for IBD, warranting further well-designed clinical trials to fully assess their therapeutic potential.

## Introduction and background

Given the prevalence of inflammatory gastrointestinal (GI) conditions worldwide, understanding and learning how to take advantage of the anti-inflammatory properties of Ayurvedic herbs and dietary recommendations may provide more long-term benefits with fewer side effects than current conventional methods. There is a limited body of peer-reviewed studies with consolidated evidence regarding the efficacy of Ayurvedic eating patterns and herbs on inflammatory conditions of the GI system in adults. This review will not explore other aspects of Ayurvedic intervention, such as mental health, physical activity practices, and individualized treatment based on dosha type. 

Ayurveda is a system of medicine that originated in India over 3,000 years ago, which focuses on the balance of the mind, body, and spirit. Ayurveda focuses on longevity and disease prevention, utilizing the body’s innate healing ability [1]. Within Ayurveda, disease processes and inflammation are thought to be a result of the imbalance of the life forces, also called doshas. The three doshas include vata, pitta, and kapha, and a particular ratio of each of these is found within the body, which differs from person to person [2]. The imbalance of these doshas can lead to particular health ailments, for which Ayurveda emphasizes altering diet (*Ahar*) and food (*Anna*) to maintain or reestablish the balance of one’s dosha. Foods are not considered inherently *good* or *bad* in Ayurveda, but rather all foods are considered to have either an aggravating or pacifying action on each dosha type based on their unique morphological features and physiological effects [3,4]. Some examples of general recommendations for healthy eating patterns include: eating freshly cooked warm meals, utilizing herbs and spices to balance the foods in a meal, avoiding antagonistic combinations such as milk with bananas, as well as eating with a relaxed, calm, and present mind [1]. 

Pitta dosha, a fundamental concept in Ayurveda, plays a crucial role in digestion and metabolic processes [5]. It is known as the heat energy in the body, and it controls most of the digestive processes [6]. According to Ayurveda, pitta dosha primarily resides in the liver, which is an organ essential for facilitating digestion, metabolism, and nutrient absorption as it governs the *jatharagni*(digestive fire), and in turn, the mechanisms of digestion [7]. An impaired pitta dosha might relocate in the body, for example, to the muscle and flesh, where it manifests as ulcers, skin conditions, and fever, which are conditions that pitta-dominant individuals are relatively prone to [8,9]. Beyond dosha dominance and susceptibility, another pathological factor that causes disease is called *Ama*, an umbrella term for undigested metabolic waste, which becomes toxic to the body in large quantities. Impaired digestion can lead to the buildup of *Ama*, resulting in the disturbance of all three doshas, leading to inflammation and disease [10]. 

Herbs and spices also play an important role in the Ayurvedic diet recommendations, and they function not only to enhance flavor but also to bring balance to the food with their ability to reduce inflammation, modulate the gut microbiome, and protect from infection. Ginger, for example, will not only help to balance the heavy properties of a food such as pork, but it can also be used to help treat ailments such as indigestion, vomiting, cold, and cough [1]. The herbs slippery elm and licorice, as well as a widely known polyherbal medicinal formulation (common name: triphala), were found to have significant prebiotic properties and therefore a profound impact on the composition of the gut microbiota [11]. Ayurveda utilizes many medicinal herbs for the treatment of disease. *Guggulutikthaka gritha* is another herbal medicine used as a remedy for chronic inflammatory conditions, and research has shown its efficacy in its ability to control hyperglycemia, hyperlipidemia, and inflammation in rats with known dyslipidemia [12]. In addition, certain GI inflammatory conditions, like peptic ulcer disease, are mainly caused by an imbalance in the offensive and defensive factors of the gut mucosal epithelium and are treated with herbs like *Acacia arabica* to restore the balance [13,14]. Due to the various side effects resulting from conventional drug usage, many medicinal plants offer an alternative, natural approach to healing GI inflammatory conditions [14].

Inflammatory conditions of the GI tract are prevalent worldwide and encompass various subsets of disorders. Inflammatory bowel disease (IBD) is a term that includes conditions such as ulcerative colitis (UC) and Crohn’s disease (CD). UC is characterized by inflammation and ulcerations in the mucosa and submucosa found in the large intestine and often presents with crypt abscesses in a continuous pattern ​​[15]. Conversely, CD exhibits transmural inflammation, ulcerations in a non-continuous pattern, and typically affects the terminal ileum and cecum, but can be found anywhere along the GI tract. IBD represents a chronic inflammatory disorder marked by recurring episodes of intestinal inflammation and periods of remission. While the precise etiology of IBD remains unclear, it is evident that genetic factors, microbiota composition, environmental factors, and immunological dysregulation all play a role in its occurrence [15]. IBD shows a consistently increasing global incidence since 1990 for both men and women and was estimated to affect approximately 4.9 million people in 2019 [16]. In 2016, the overall annual healthcare spending on IBD was estimated to be over $25 billion in the United States alone [17]. 

Various approaches are used for managing and treating IBD, but there is still ongoing research on this topic. Currently, IBD symptoms are primarily treated with corticosteroids, aminosalicylates, and immunosuppressants [18]. In patients who do not tolerate conventional treatments, alternative drugs such as calcineurin inhibitors, tumor necrosis factor-α (TNF-α) inhibitors, and targeted antibiotics may be prescribed. Surgical intervention, including colon resection, is typically considered when pharmacological treatments are ineffective or when complications arise, such as high-grade dysplasia, IBD-associated colorectal cancer, or severe disease unresponsive to medical therapy [18]. However, it is important to note that these medications may not consistently provide satisfactory relief [19]. The primary aim of most conventional Western medicine is targeted towards alleviating symptoms rather than addressing the underlying root cause [18].

Beyond pharmaceutical interventions, there is ongoing research investigating nutritional strategies to modulate the immune response in IBD. Dietary choices have a significant impact on gut health. The Western diet, characterized by an abundance of fried and processed foods, promotes the release of pro-inflammatory cytokines and disrupts the gut microbiome, contributing to a variety of GI inflammatory conditions [20]. Certain diets have shown promising results in addressing IBD symptoms. A low FODMAP (fermentable oligosaccharides, disaccharides, monosaccharides, and polyols) diet, for instance, has been associated with symptom relief, including reducing bloating, diarrhea, nausea, and fatigue [20]. In addition, the Mediterranean diet, which is known for its anti-inflammatory properties, was seen to be beneficial during remission phases, although it may not be suited for patients experiencing active episodes due to its high fiber content [20].

While the literature on nutritional interventions for IBD is being established, there are still several existing gaps in Ayurveda research, especially about the efficacy of Ayurvedic herbs on diseases of the GI tract. Broadening knowledge in this sector can help educate patients and inform practitioners on how diet and the use of Ayurvedic herbs can address dosha imbalances and help treat various diseases. This can lead to long-term relief and improvement of symptoms with minimal to no side effects, like no increased risk of infection or occult blood post-treatment, when ingested correctly [18]. This paper provides a scoping review of the existing literature regarding beneficial Ayurvedic herbs to prevent and treat inflammatory GI conditions. This review will not explore other aspects of Ayurvedic intervention, such as mental health, physical activity practices, and individualized treatment based on dosha type. 

## Review

Methodology

Eligibility Criteria

To meet the inclusion criteria for this review, articles had to be peer-reviewed and based on original research studies published in English from January 2003 to September 2023. Both human and animal studies involving the method of action of Ayurvedic herbs as they relate to the colon and inflammatory processes, as well as human studies on the outcomes of Ayurvedic treatment for IBD, were included. We expanded our search to include articles both in the United States and worldwide due to limited research in Ayurveda in general and the increasing concern of IBD worldwide.. Also, research studies on Ayurveda are limited, and we wanted to expand our search. Studies that included child participants, in vitro studies, dissertations, books, recommendations, or opinions, and those not available as full text were excluded.

Both experimental and quasi-experimental study designs, including randomized controlled trials, non-randomized controlled trials, before and after studies, and interrupted time-series studies, were considered for inclusion. Analytical observational studies, including prospective and retrospective cohort studies, case-control studies, and analytical cross-sectional studies, were also considered. Finally, we looked at descriptive observational study designs, including case series, individual case reports, and descriptive cross-sectional studies. Our review was specifically focused on the utilization and effectiveness of Ayurvedic herbs in the management of IBD in the United States and worldwide. 

Information Sources

This scoping review utilized the following three electronic databases: Embase, PubMed, and Cochrane Central. A comprehensive search was done to include published as well as difficult-to-locate or unpublished studies. The search strategy was limited to any peer-reviewed original research articles published in English from January 2003 to September 2023. The literature search was last executed on November 19, 2023, and articles were also hand-picked from the reference lists of the included sources of evidence. 

**Figure 1:**
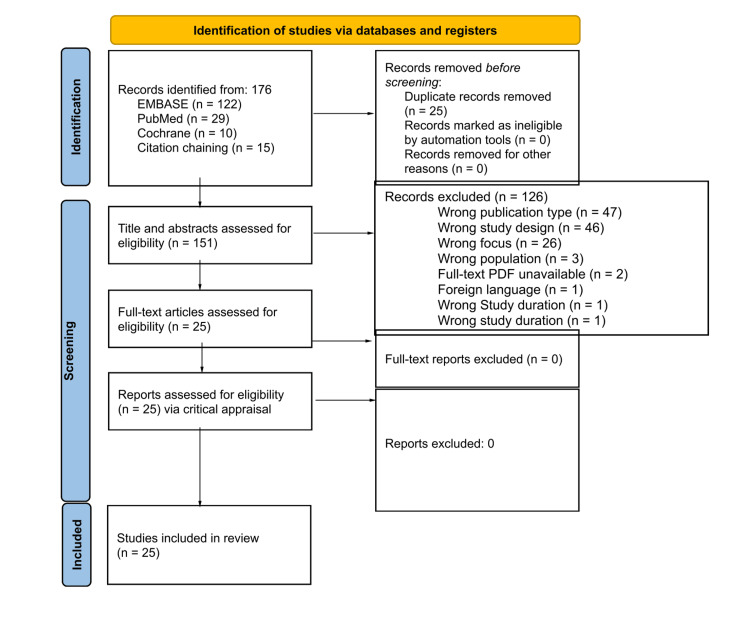
PRISMA 2020 flow diagram for new systematic reviews that included searches of databases and registers only. PRISMA, Preferred Reporting Items for Systematic Reviews and Meta-Analyses

The search identified a total of 176 articles. Twenty-five duplicate articles were removed, so 151 titles and abstracts were assessed for eligibility. A total of 126 articles were excluded due to wrong publication type (*n* = 47), wrong study design (*n* = 46), wrong focus (*n* = 26), wrong population (*n* = 3), unavailability of the full-text PDF (*n* = 2), foreign language (*n* = 1), and wrong study duration (*n* = 1). Twenty-five full-text articles were assessed for eligibility and retained for analysis. The screening and selection process is depicted using a Preferred Reporting Items for Systematic Reviews and Meta-Analyses (PRISMA) flowchart in Figure 1. 

Search Strategy 

The search question was based on the Population, Concept, and Context (PPC) strategy. The population of adults with confirmed inflammatory conditions of the gastrointestinal system or animal studies with induced IBD, the concept of analyzing the efficacy of Ayurvedic herbs in the treatment and prevention of IBD, and the context of traditional Ayurvedic lifestyle for IBD worldwide were established. The guiding question for this research was *How do Ayurvedic herbs aid in the treatment and prevention of inflammatory bowel diseases? *Data collection took place in November 2023, and the initial limited search of Embase, PubMed, and Cochrane Central was conducted. 

Two co-authors, both third-year medical students, conducted the initial literature search for this review. The text words contained in the titles and abstracts of relevant articles, and the index terms used to describe the articles, were used to develop a full search strategy for three databases (Embase, PubMed, and Cochrane Central ). The following defined search terms were used: “Ayurveda” OR “Ayurvedic Medicine” OR “Ayurvedic Herbs” OR “Ayurvedic Nutrition” OR “Ayurvedic Diet” OR “Inflammatory Bowel Disease” OR “Ulcerative Colitis” OR “Crohn’s Disease”.

The search strategy, including all identified keywords and index terms, was adapted for each included database and/or information source. The reference list of all included sources of evidence was screened for additional studies. Studies published only in the English language were included. Studies published between 2003 and 2023 were included to find the most updated and relevant information.

A total of 161 articles were identified in the three databases, and 15 were added in the search based on the reference analyzed. The PRISMA method was adopted to systematize the inclusion process of the studies.

Selection of Sources of Evidence

The first and the second authors reviewed the titles and abstracts of 176 articles of the initial search for the eligibility criteria and duplication. Twenty-five articles were deleted due to duplication. The faculty mentor was the tiebreaker for any disagreements. Articles were excluded due to wrong publication type (*n* = 47), wrong study design (*n* = 46), wrong focus (*n* = 26), wrong population (*n* = 3), unavailability of the full-text PDF (n = 2). foreign language (*n* = 1), wrong study duration (*n* = 1). Selected 25 full-text articles were then reviewed by the fourth and eleventh authors. The eighth author was assigned as the tie breaker, but there was no conflict with these selections. 

Data Charting Process

The 25 articles that passed three levels of screening for inclusion in our scoping review underwent data extraction. A data-charting form was created by two reviewers to determine which variables to extract. The research articles were split among the ten authors. Each author reviewed two or three articles, and the data were independently charted into a standardized chart in Microsoft Excel. 

Data Items

Data were compiled in an Excel spreadsheet based on article characteristics (including year of publication, study type, origin, population, methods), and information relevant to our review question (including aims, key findings, limitations, and take-away points).

Critical Appraisal of Individual Sources of Evidence (Quality Appraisal)

The quality and validity of an article are paramount in the article selection process because the results and data produced by articles could be biased or skewed, which, in turn, will affect the validity of this current scoping review. For this reason, a systemized and thorough review of all semi-finalist articles after tier-two screening was performed. The process of screening included using a critical appraisal tool. The critical appraisal tool used in this review is the Joanna Briggs Institute Critical Appraisal Tools (JBI). This appraisal tool is a trustworthy tool that works to constantly improve and offers an effective approach to evaluating the quality of an article. The checklist provided by JBI takes into account the research biases, overall congruence, and imperative sections that all contribute to the quality of an article. The tools allowed for the classification of the articles as high risk of bias (scores less than 50%), moderate risk of bias (scores between 50% and 70%), and low risk of bias (scores above 70%). Two researchers then read all semifinalist articles in-depth and blindly appraised the articles using the applicable JBI appraisal tools. This appraisal process was then followed by deliberation between two researchers who compared and contrasted their appraisal scores. A third researcher served as a tie-breaker for any discrepancies in the appraisal scores. The relevancy and quality of each article were discussed in great detail between the researchers, and a final consensus was reached, where the final articles were chosen. Three articles were excluded for various reasons. The first was excluded due to a lack of a placebo group and being non-blinded. The second utilized self-reporting, which limited its reliability and had broad inclusion criteria. The third also contained broad inclusion criteria as well as confounding factors that were not properly controlled. Additionally, several articles were limited by their use of animal subjects as opposed to human subjects. However, they were ultimately included due to their robust methodology and implications for human application.

Synthesis of the Results

The final articles were grouped by the different Ayurvedic herbs (curcumin, triphala, Boswellia serrata, etc.) and polyherbal formulations. The results were summarized in a table format in Excel, which includes the range of evidence that was found from the review. 

Results

Figure 1 details the PRISMA flowchart, which describes the search process conducted for this scoping review. There were a total of 176 records obtained originally. A total of 122 articles were identified from Embase, 29 from PubMed, 10 from Cochrane, and 15 from Citation Chaining. Twenty-five duplicate records were removed. Then, a total of 151 titles and abstracts were assessed for eligibility. Out of these articles, 126 records were excluded due to various reasons, like having the wrong study design, wrong focus, wrong population, wrong study duration, unavailability of the full-text PDF, or being the wrong publication type. These exclusions were all performed during the tier 1 and tier 2 selection processes. Both of these were performed with two reviewers and one tiebreaker. Twenty-five full-text articles were assessed for eligibility via the critical appraisal process and included in the review.

Characteristics of the Reviewed Studies

The intervention type for most (*n* = 23) of the articles that were selected for this study administered a single drug or a formulation with several drugs to humans (*n *= 8) or animal models (*n *= 15). Two of the articles were questionnaires about complementary and alternative medicine (CAM). The most frequent duration of the studies was 7-20 days (*n *= 12). Other studies in the literature were conducted for approximately one month (*n *= 8). Additionally, some studies were conducted for a longer duration, in which three lasted 12-14 weeks [21-23] and the remaining three lasted for around one year [24-26]. 

The population of the studies included a mix of human studies (adult males and females with IBD) [22,25-32] and animal studies [21,33-46], while two were CAM surveys/questionnaires [23,24]. The studies were conducted in various countries, with a majority of studies from India (*n* = 14), while the others were from South Korea (*n* = 1), Egypt (*n* = 1), various parts of Europe (*n* = 8), and Iran (*n* =1). 

The studies aim to assess the usage of a variety of herbs concerning IBD. The most frequent observation was using a particular plant extract and its effect on IBD (*n* = 16). Three of the studies focused on the use of curcuminoids, while two tested the effects of *Boswellia serrata*. Other treatment modalities included herbal preparations with a combination of ingredients (*n* = 6), bilberry fruit (*n* = 1), and CAM (*n* = 2). The characteristics of the included studies are summarized in Table 1.

**Table 1:** Summary table of the characteristics of the included studies. CAM, complementary and alternative medicine

Characteristic	Details
Type of study	Drug/formulation in humans (*n *= 8), drug/formulation in animals (*n *= 15), CAM surveys (*n *= 2)
Study duration	7-20 days (*n *= 12), ~1 month (*n *= 8), 12-14 weeks (*n *= 3), ~1 year (*n *= 3)
Population	Humans with IBD (*n *= 8), animal models (*n *= 15), CAM questionnaire respondents (*n *= 2)
Geographic distribution	India (*n *= 14), Europe (*n *= 8), South Korea (*n *= 1), Egypt (*n *= 1), Iran (*n *= 1)
Type of intervention	Single plant extract (*n *= 16), Curcuminoids (*n *= 3), *Boswellia serrata* (*n *= 2), combination of herbal formulas (*n *= 6), bilberry fruit (*n *= 1), CAM (*n *= 2)

Quality of the Studies

The methodologies of the 25 included articles varied on a wide spectrum. Multiple methodologies were used in the included articles, including exploratory analysis [26], surveys [24], cross-sectional studies [23], an open, prospective, non-blinded, and non-controlled pilot trial [25], and an open, non-randomized, monocentric clinical trial [27]. Controlled randomized trials were observed to be a common methodology among the 10 different studies. Six studies followed a variation of randomized trials, and the remaining five adopted non-specified methodologies. The included studies met the inclusion criteria of the review. 

Anti-inflammatory Properties

Inflammation in the body is a major factor in the progression of IBD. Several studies utilized inflammatory markers to give insight into the progression of IBD [21,25,31,33,37,38,45]. TNF-α and IL-6 are key pro-inflammatory cytokines that are more prominently associated with CD. The studies demonstrated a selection of Ayurvedic treatment and their efficacy in reducing these inflammatory biomarkers. Herbs such as *Sesbania grandiflora* demonstrated a quantified decrease in the levels of TNF-α by 3.97% and IL-6 by 15.89% in mice with acetic acid-induced UC [38]. Another study illustrated that the use of the methanol fraction of *Cordia dichotoma* bark significantly decreased the production of both TNF-α and PGE2. These treatment groups also showed good healing and less infiltration by neutrophils and other inflammatory cells [21]. In addition, one study demonstrated how the hemoglobin increased and inflammatory markers like ESR decreased with the administration of polyherbal ayurvedic formulations [31]. A polyherbal formulation of certain potent Ayurvedic herbs like *Eryngium foetidum* (Burma dhaniya), *Manilkara zapota* (Sapota), and *Murraya koenigii* (curry leaves) reduced signs of inflammation by decreasing myeloperoxidase (MPO) activity through its antioxidant components like alkaloids and flavonoids [37]. *Terminalia arjuna* hydro-alcoholic extract administration relieved the disease activity in 2,4,6-trinitrobenzene sulfonic acid (TNBS)-induced colitis by reducing the expression of pro-inflammatory cytokines and chemokines like MPO, malondialdehyde, and nitric oxide, decreasing oxidative stress, and improving plasma zinc level and structure of gut microbiota [33].

Curcumin, found in turmeric, is a natural anti-inflammatory product that provides a nontoxic alternative to modulate inflammatory disorders. Two studies explored the effect of curcumin as a potential treatment for IBD. In one study, curcumin pretreatment resulted in marked suppression of inflammatory markers like Interferon-gamma (IFN-g), IL-12, p40 mRNA levels, and iNOS mRNA expression with a little induction of IL-4 mRNA in TNBS-treated mice [45]. Another study found that the use of curcuminoids led to significant improvement of symptoms such as reduced frequency of urgent defecation, improved patients' self-reported well-being, and reduced clinical activity of UC [30]. 

While reduction in inflammation is a major component of treating IBD, some Ayurvedic extracts also demonstrated an added protective effect against mucosal damage. One study looked at the effect of *Canna x generalis* rhizome ethanol extract (CGE) on dextran sulfate sodium (DSS)-induced UC in mice. Administration of CGE (100 mg/kg) caused a marked reduction in NF-ҡB expression compared to the DSS-induced group without CGE. Furthermore, CGE was also associated with a marked reduction in caspase-3 expression, an enzyme normally responsible for increased apoptosis of colon epithelial cells [43]. In addition to lowering the inflammatory markers, this herbal extract might preserve and repair the mucosal tissue in patients with IBD. 

Many studies compared the effect of the Ayurvedic treatment with the mainstream corticosteroid treatment of prednisolone and sulfasalazine. Prednisolone is a delayed-release corticosteroid that has anti-inflammatory as well as immunosuppressive effects. Sulfasalazine is a disease-modifying anti-rheumatic drug (DMARD) that also has anti-inflammatory properties. Some polyherbal formulations demonstrated a comparable effect to prednisolone, such that both treatment modalities resulted in a similar decrease in MPO activity in rat and mouse models [36,37,39]. An Ayurvedic polyherbal formulation at a dose of 275 and 550 mg/kg produced similar protective effects for colitis as 60 mg/kg of prednisolone. In addition, *Amorphophallus paeoniifolius* extract, a plant native to Southeast Asia, is effective in reducing UC symptoms, and its efficacy is comparable to that of prednisolone. *Amorphophallus paeoniifolius* revealed the potential to surpass traditional UC treatments such as prednisolone, as prednisolone also has long-term side effects that negatively impact patients’ lives [36]. In a study that examined the effects of gum resin of *Boswellia serrata *in treating patients with chronic colitis, it was found that there were comparable effects in lowering abdominal pain, improving stool properties, lowering inflammation, treating ulcerations, and increasing blood parameters for both the experimental group treated with sulfasalazine and the Boswellia gum resin group, both of which had significant improvements over the control group [27]. While one study looking at the use of a different herb,* Sesbania grandiflora*, in acetic acid induced UC rats revealed prednisolone to be more effective than that herbal source, the herbal treatment still yielded statistically significant results in comparison to the placebo. Treatment with *Sesbania grandiflora* decreased the IL-6 levels by 3.97% (100 mg/kg) and 15.89% (200 mg/kg), while the use of 2 mg/kg prednisolone decreased IL-6 by 26.49% [38]. 

While microscopic effects of treatment in IBD are measured by inflammatory markers, other studies used macroscopic measures such as mucus in stool, bowel frequency and consistency, and occult blood [28,35,40]. A mono-herbal study with *Orocylum indicum* in rats revealed a statistically significant reduction in gross damage area, weight loss, and an increase in colon weight in the substance-pretreated animals in comparison to the control group. These results were consistent with a microscopic comparison of the same groups with a reduction in MPO activity, malondialdehyde levels, and nitric oxide levels, and increased glutathione levels [40]. 

Histopathological Changes

Histopathological changes reflect the status and progression of a disease with cell morphological changes. Some of the Ayurvedic drugs used revealed significant decreases in inflammation on histology that were comparable to or greater than the traditional allopathic medicine used for these conditions [27,37]. The use of a polyherbal formulation with certain potent Ayurvedic herbs like *Eryngium foetidum* (Burma dhaniya), *Manilkara zapota* (Sapota), and *Murraya* *koenigii* (curry leaves) resulted in lessened damage seen on the histopathology, which reflected the decrease in inflammation and painful symptoms, compared to the control group [37]. These results were comparable to the Prednisolone effects seen in the same study [37]. In a study examining the effects of gum resin of* Boswellia serrata* in treating patients with chronic colitis, both the Boswellia-treated group and the sulfasalazine group experienced comparable symptom relief. However, qualitative assessments of histopathology and scanning electron microscopy revealed more pronounced improvements in the Boswellia group, including reduced mucosal inflammation and tissue damage, with minimal side effects [27].

Improvement in Symptoms

The symptoms associated with IBD can potentially cause great discomfort in patients. With the administration of certain Ayurvedic polyherbal formulations, there were substantial improvements in symptoms such as bowel frequency, bleeding, urgency, and abdominal pain [41,30-32]. Bowel frequency was reduced by 81.81%, bleeding in stool was reduced by 91.58%, abdominal pain was reduced by 86.76%, weakness was reduced by 65.97%, and body weight increased by 2.31% in a study that gave Ayurvedic polyherbal formulations of *Udumbara kvatha basti *with oral *Kutaj ghan vati*, *Udumbara kvatha*, and a combination of *Musta, Nagakesara, Lodhra, Mukta panchamrut rasa* to the patients. All results were statistically highly significant, concluding that this concoction was a safe and effective alternative for UC patients according to this study [31]. In another study, adding curcuminoid nanomicelles to the routine treatment of patients with UC was associated with a significant improvement of symptoms, including reduced frequency of urgent defecation, improved patient self‐reported well‐being, and reduced clinical activity of UC [30]. In a study conducted in New Zealand that worked with male ICR mice and guinea pigs, Acetyl-11-keto-b-boswellic acid significantly and in a concentration-dependent manner was shown to inhibit the contractile response elicited by acetylcholine, which led to a reduction in symptoms [32].

CAM use among patients in the United Kingdom and France was analyzed by sending anonymous questionnaires and surveys to patients [24,23]. This study was conducted to understand the prevalence of herbal or alternative medicine used there to maintain general health in a small population set in Scotland, United Kingdom. The study explained that many people (60%) in research use CAM to influence their health, so healthcare professionals need to have more knowledge about these supplements. This study also called for more research on CAM so the general population does not harm themselves using supplements with safety profiles that still need to be well-researched [23]. In France, the survey results indicated that the rates of good quality of life (SIBDQ > 50) were higher in the CAM user group. In addition, patients reported an improvement in their quality of life with CAM use [24].

Outcomes

While most studies reported improvements in one or more factors associated with Ayurvedic treatment, one trial did not show significant benefits [29]. The study examined the efficacy and safety of aloe vera gel in treating mildly to moderately active UC. Although the herbal extract appeared to reduce both clinical and histological disease activity scores, the symptomatic improvement did not achieve statistical significance compared to placebo. In this randomized trial, 44 hospital outpatients were randomly assigned to receive either oral aloe vera gel or placebo (100 mL twice daily for four weeks) in a 2:1 ratio. However, due to the limited sample size, clinical remission and improvement rates with aloe vera gel failed to achieve statistical significance [29]. Although this negative outcome may be due to the relatively small sample size, it should be highlighted that aloe vera is just one of many Ayurvedic herbs, and it could have a lower efficacy potential in comparison to the other herbs mentioned in this scoping review. While the majority of studies mentioned in this scoping review produced a positive outcome in the disease progression, further exploration is warranted to further evaluate the use of Ayurvedic herbs as a complementary or alternative treatment in IBD. 

Discussion

Principle Findings

Ayurveda is an ancient medicinal system with its roots in India. Ayurveda attributes disease and inflammation to imbalances in the body's life forces, or doshas: vata, pitta, and kapha, each of which varies uniquely across individuals. Imbalances in these doshas can cause specific health issues, prompting Ayurveda to emphasize dietary and lifestyle adjustments to restore balance [3,4]. Ayurvedic medicine aims to support the body’s innate healing mechanisms by promoting balance and overall well-being. Ayurvedic medicine believes food is medicine, and natural herbs and extracts, if utilized properly, can help restore the body’s imbalances [46]. This scoping review aimed to systematically examine the existing literature on the efficacy of Ayurvedic herbs in the context of IBD. The findings from the reviews provided valuable insights into the potential of various Ayurvedic herbs and polyherbal formulas to be utilized as complementary or alternative interventions for managing IBD. 

The available evidence revealed the diversity of Ayurvedic herbs studied and their impact on IBD. The included studies investigated a wide range of herbs, such as turmeric (Curcuminoid), boswellia (*Boswellia serrata*), and many other formulations. These herbs were shown to have anti-inflammatory, antioxidant, and immunomodulatory properties, which are crucial aspects of distress and imbalance in the context of IBD pathogenesis [27,40,45]. Furthermore, certain studies demonstrated comparable effects of ayurvedic herbs in decreasing inflammatory markers like MPO activity and IL-6, as well as symptom relief when compared to conventional medications like prednisone and sulfasalazine, with minimal side effects [27,36]. These statistically significant results suggest the potential use of Ayurvedic medicine in the management of IBD to target the root cause of inflammation and improve symptoms effectively and naturally.

The main objective of this scoping review was to investigate the efficacy of ayurvedic herbs on IBD. After an extensive process of review and filtering, 25 articles were isolated for analysis. Most of these articles targeted a mono-herbal or ayurvedic polyherbal formula utilizing an extract and comparing it to a placebo group or traditional modern medicine to evaluate the results of administering an Ayurvedic treatment (*n* = 16). Whether it was by enhancing the body’s anti-inflammatory properties, inducing histopathological changes, or improving symptoms associated with IBD, most studies were associated with statistically significant positive outcomes (n=24). Not only did they reveal reduced inflammation, but they accomplished this with minimal side effects as compared to their conventional medicine counterparts [36]. One study did not result in statistically significant positive outcomes from an Ayurvedic herbal treatment. This study investigated an aloe vera extract and demonstrated a reduction in both clinical and histological disease activity scores, leading to a more notable symptomatic improvement compared to placebo. However, this response did not reach statistical significance, which could have been due to a relatively smaller size or the efficacy potential of this specific herb, which is just one of the many countless Ayurvedic herbs [29]. Even though Ayurvedic medicine has a scope and potential to be used more widely as complementary or alternative medicine, large voids in knowledge do exist, so more extensive studies have to be conducted to properly evaluate the safety profiles, interactions, and appropriate dosages of the herbal formulations.

Ayurvedic medicine has been practiced for thousands of years, but more clinical trials need to be conducted to understand the efficacy and proper usage of Ayurvedic herbal treatment through the modern medicine lens. Ayurvedic knowledge has been traditionally passed on through many generations; however, this ancient medicinal practice lacks appropriate evidence-based backing due to scarce trial-based clinical research in this field. Since there are limited available research articles, this scoping review plays a crucial role in aggregating the existing information in the context of treating IBD with Ayurveda. 

Ayurvedic Medicine in the Western World

Allopathic treatment aims to treat symptomatically, while Ayurvedic medicine leans more toward holistic care by emphasizing treating the root cause of the ailment with lifestyle and food modifications. For overall health maintenance, they emphasize the importance of taking care of the spiritual, physical, and mental health of a patient [47]. Currently, within allopathic medicine, there is not a single effective treatment for the eradication of IBD, and many treatment options result in undesirable side effects [33]. One mainstream treatment involves the use of prednisone to decrease the inflammation caused by IBD. While this treatment is known to be an effective steroidal anti-inflammatory, long-term use of prednisone results in many adverse side effects, including hyperglycemia, insomnia, increased appetite, osteoporosis, and edema [48]. The Ayurvedic approach gives various treatment options, with fewer side effects, that may be beneficial to the whole body rather than targeting only symptomatic relief [21,24,39,42].

Limitations

Ayurvedic medicine can be a powerful tool in managing inflammatory conditions because many aspects of Ayurveda aim to heal inflammation. However, conducting clinically based research on proper Ayurvedic medicine can be difficult due to numerous reasons. Many of the clinical trials explored in this study did not include treatment delivered by an Ayurvedic doctor. Moreover, most of the treatments in the studies used a *one-size-fits-all *formula, whereas actual Ayurveda focuses on individualized care based on patients’ symptoms and body type. Also, the studies did not showcase a comprehensive Ayurvedic diagnosis and treatment that encompasses lifestyle, food quantity and composition, and spiritual practice changes, but rather studied the effects of herb extracts on clinical symptoms or modulation of inflammatory markers. 

Gaps in the Research and Areas of Further Research

Several gaps surfaced during our investigation, particularly in the exclusion of patients with severe UC or other complications from certain studies [22,30,31,41]. This limitation may impact the overall applicability of the results to a broader population of patients worldwide. Since Ayurvedic medicine is still a relatively new approach in Western society, most of the studies have been conducted in the Eastern part of the world. Many studies shared similar limitations and mainly looked at the response to treatment rather than the mechanism of action for the Ayurvedic approach. Additionally, many studies predominantly assessed the effects of Ayurvedic treatments on animal models rather than human subjects [21,32-40,42,43,45]. While animal models share similarities in inflammatory marker responses, they cannot precisely replicate the impact of Ayurvedic treatments on the human body. Even when assessing human subjects, the gut microbiota composition may vary for humans in different countries and seasons. Therefore, the geographic location of the patients, the accessibility to certain herbs, and the interactions with the common diet are factors that need to be accounted for in future studies before deeming genuine Ayurvedic treatment acceptable as a parallel therapeutic approach.

Finally, a common limitation identified across most trials was the relatively small sample sizes and short durations, often lasting around one month or less [21,27,29,37,38,39,41,43]. Randomized controlled trials with larger sample sizes and longer follow-up periods are essential to establish causation, long-term side effects, complications, sustained positive or negative effects, and potential interactions to determine the long-term safety and efficacy of Ayurvedic herbs in managing IBD. 

Going forward, it is essential to recognize that Ayurveda extends beyond merely using herbs to treat existing ailments; it encompasses a holistic way of life. Our findings emphasize the need for further research into Ayurvedic principles outlined in ancient texts, exploring dietary restrictions, eating patterns, ideal consumption quantities, recommended food types, and overall lifestyle practices for maintaining optimal wellness. Furthermore, collaboration between allopathic or osteopathic medical physicians and Ayurvedic practitioners could facilitate the development of research methodologies that integrate the strengths of modern medicine and Ayurvedic knowledge.

This scoping review provides a comprehensive overview of the current state of evidence regarding the efficacy of Ayurvedic herbs in IBD management. While promising findings suggest the potential benefits of Ayurvedic herbs as complementary or alternative medicine, the limitations of existing studies highlight the need for further research. Well-designed clinical trials worldwide, standardization of interventions, evaluation of safety profiles of herbs, and collaboration between traditional and conventional medicine communities are essential steps going forward. The integration of Ayurvedic medicine into mainstream healthcare for IBD management warrants continued exploration.

## Conclusions

A scoping review was conducted that included 25 full-text articles to understand the extent of the literature surrounding the use of Ayurvedic herbs to help treat IBD. Most of the studies showed statistically significant improvements in inflammation with the use of a single extract or using a combination of multiple extracts (*n* = 24). The studies revealed the anti-inflammatory properties, histopathological changes, and improved symptoms associated with the use of various Ayurvedic herbs on the pathogenesis of IBD. Even though there are limited clinical trials in this field of research, this scoping review showcases the knowledge from existing literature on the efficacy of Ayurvedic herbs in preventing and treating gastrointestinal inflammatory conditions. Finally, this review calls for further clinical trials in the study of Ayurveda to potentially incorporate Ayurvedic treatment into modern medicine as complementary or alternative interventions for managing gastrointestinal inflammatory conditions, like IBD, to help heal the whole body with limited side effects.
